# Application of remimazolam anesthesia in elderly patients undergoing radical resection for colorectal cancer: a cohort study on gastrointestinal recovery and complication rates

**DOI:** 10.3389/fonc.2026.1867238

**Published:** 2026-07-08

**Authors:** Yanming Xue, Jie Zhang

**Affiliations:** Department of Anesthesiology, Yantaishan Hospital, Yantai, Shandong, China

**Keywords:** colorectal cancer surgery, enhanced recovery after surgery, gastrointestinal function, postoperative complications, remimazolam

## Abstract

**Background:**

Optimizing anesthesia within the enhanced recovery after surgery (ERAS) protocol was crucial for elderly patients undergoing radical resection for colorectal cancer. This study aimed to assess the impact of remimazolam versus propofol anesthesia on postoperative gastrointestinal function recovery and complication rates in this population.

**Methods:**

This prospective cohort study included elderly patients who underwent radical cancer resection from January 2023 to January 2025. Patients were assigned to either the remimazolam group or the propofol group based on the anesthetic used. Gastrointestinal hormones were measured preoperatively and 24 hours postoperatively. Recovery times for bowel sounds, first flatus, first defecation, and first solid food intake were recorded. Postoperative complications, including nausea/vomiting and delirium, were monitored, and recovery quality was assessed using the QoR-15 scale one week postoperatively.

**Results:**

After propensity score matching, 243 patients were analyzed (remimazolam: n=115; propofol: n=128). At 24 hours postoperatively, motilin (81.47 ± 5.13 pg/mL vs. 79.61 ± 5.62 pg/mL, P = 0.008) and gastrin levels (48.25 ± 5.88 pg/mL vs. 42.72 ± 6.41 pg/mL) were higher in the remimazolam group. The remimazolam group showed shorter gastrointestinal recovery times (all P<0.05). Nausea/vomiting (5.22% vs. 13.28%, P = 0.032) and delirium (10.43% vs. 21.88%, P = 0.016) rates were lower, and QoR-15 scores were higher (125.44 ± 9.95 vs. 119.89 ± 12.82, P<0.001) compared to the propofol group.

**Conclusion:**

For elderly patients undergoing colorectal cancer surgery within an ERAS pathway, remimazolam anesthesia was associated with shorter gastrointestinal recovery times, lower observed rates of selected postoperative complications, and higher QoR-15 scores compared with propofol. Given the prospective cohort design, these findings should be interpreted as associations rather than evidence of causality and require confirmation in randomized studies.

## 1 Introduction

Colorectal cancer (CRC), characterized by the abnormal growth of cells in the colon or rectum, was a significant global health burden (1, 2). The primary treatment method was radical resection. Clinical manifestations included changes in bowel habits, rectal bleeding, and abdominal pain (3). In recent years, the focus of treatment strategies has been on improving patient outcomes through enhanced recovery after surgery (ERAS) protocols (4). Despite these advances, challenges remained in optimizing perioperative care, especially for elderly patients who often had multiple comorbidities, making anesthesia management and postoperative recovery more complex (5).

In this context, the implementation of the ERAS protocol aims to reduce surgical stress and promote patient recovery through multimodal interventions (6). Among these, the choice of anesthesia strategy, particularly the rational use of anesthetic drugs, has a critical impact on postoperative recovery outcomes. Propofol was the most widely used intravenous general anesthetic at the time (7). However, its use in elderly patients presented some drawbacks, including dose-dependent cardiovascular suppression and potential associations with postoperative nausea and vomiting (PONV) and cognitive dysfunction (8, 9). These concerns highlighted the necessity to explore potentially better anesthesia alternatives for the growing elderly surgical population.

Remimazolam, as a novel benzodiazepine, showed promise due to its rapid onset and quick metabolism, potentially reducing complications associated with prolonged sedation (10, 11). Gastrointestinal function recovery was a key determinant of postoperative recovery, especially after colorectal surgery (12). Anesthetic agents were known to influence postoperative gastrointestinal motility through complex mechanisms, which might involve the regulation of the neuroendocrine system and inflammatory responses (13). Gastrointestinal hormones played a crucial role in regulating gastric emptying and intestinal motility. Surgical stress responses and anesthetic drugs could disrupt the normal secretion of these hormones, leading to postoperative ileus (14). Therefore, understanding the role of these pathways in postoperative gastrointestinal recovery was essential for minimizing hospital stays and improving patient quality of life.

Based on the current situation, this prospective cohort study aimed to investigate the association between remimazolam anesthesia and postoperative recovery outcomes within the ERAS framework for elderly patients undergoing radical resection for CRC. The focus was on gastrointestinal function recovery indicators and the incidence of postoperative complications. The relevance of this study lies in its specific analysis of remimazolam within the ERAS context for elderly colorectal surgery, which may provide hypothesis-generating evidence for optimizing future anesthesia strategies in this vulnerable patient group.

## 2 Methods

### 2.1 Research design

This study was a prospective cohort study based on the ERAS concept. It aimed to evaluate the effectiveness of remimazolam anesthesia in elderly patients undergoing colorectal cancer resection, with an analysis of postoperative gastrointestinal function recovery and complication rates. As treatment allocation was not randomized and was based on the anesthetic used in routine clinical practice, the study findings should be interpreted as associations rather than causal effects. Data were collected from elderly patients who underwent colorectal cancer resection at Yantaishan Hospital between January 2023 and January 2025. Patients were divided into two groups based on the anesthetic used during surgery: the propofol group and the remimazolam group. A total of 141 patients received propofol anesthesia, while 134 patients received remimazolam anesthesia. To minimize the impact of baseline characteristic differences on the results, we used propensity score matching. After matching, the final sample included 128 patients in the propofol group and 115 patients in the remimazolam group.

This is a prospective cohort study. All participants provided written informed consent prior to their inclusion in the study. All data processing and analysis strictly adhered to the regulations of the Yantaishan Hospital ethics committee and received approval from that committee. We ensured the protection of patient privacy and data security throughout the study, with all data used solely for research purposes.

### 2.2 Participants

This study included patients aged 65 years or older who underwent colorectal cancer resection and met the ERAS pathway management standards (15). And had no severe cardiopulmonary dysfunction or other major comorbidities that would affect anesthesia selection, including severe uncontrolled arrhythmias, severe dyspnea at rest or requiring long-term oxygen therapy, liver cirrhosis classified as Child-Pugh grade C, persistent estimated glomerular filtration rate (eGFR) <30 mL/min/1.73m², uncontrolled severe neurological disorders, active systemic infections, or uncorrected severe coagulation disorders.

Patients were excluded if they had allergies to benzodiazepines or anesthetics, or if they exhibited severe organ dysfunction (including respiratory, circulatory, liver, or kidney systems) before surgery. Individuals on long-term sedatives or analgesics, those requiring perioperative blood transfusions, or participants in other clinical studies within the past three months were also excluded. Additionally, patients with evidence of distant metastasis during preoperative assessment, or those who received chemotherapy, radiotherapy, or corticosteroid treatment before surgery, did not meet the study criteria. Patients who could not complete remimazolam anesthesia induction or maintenance, or whose surgery needed to switch from laparoscopic to open surgery due to complications, were also excluded from the study. Patients with incomplete data were excluded from the analysis.

**Figure 1:**
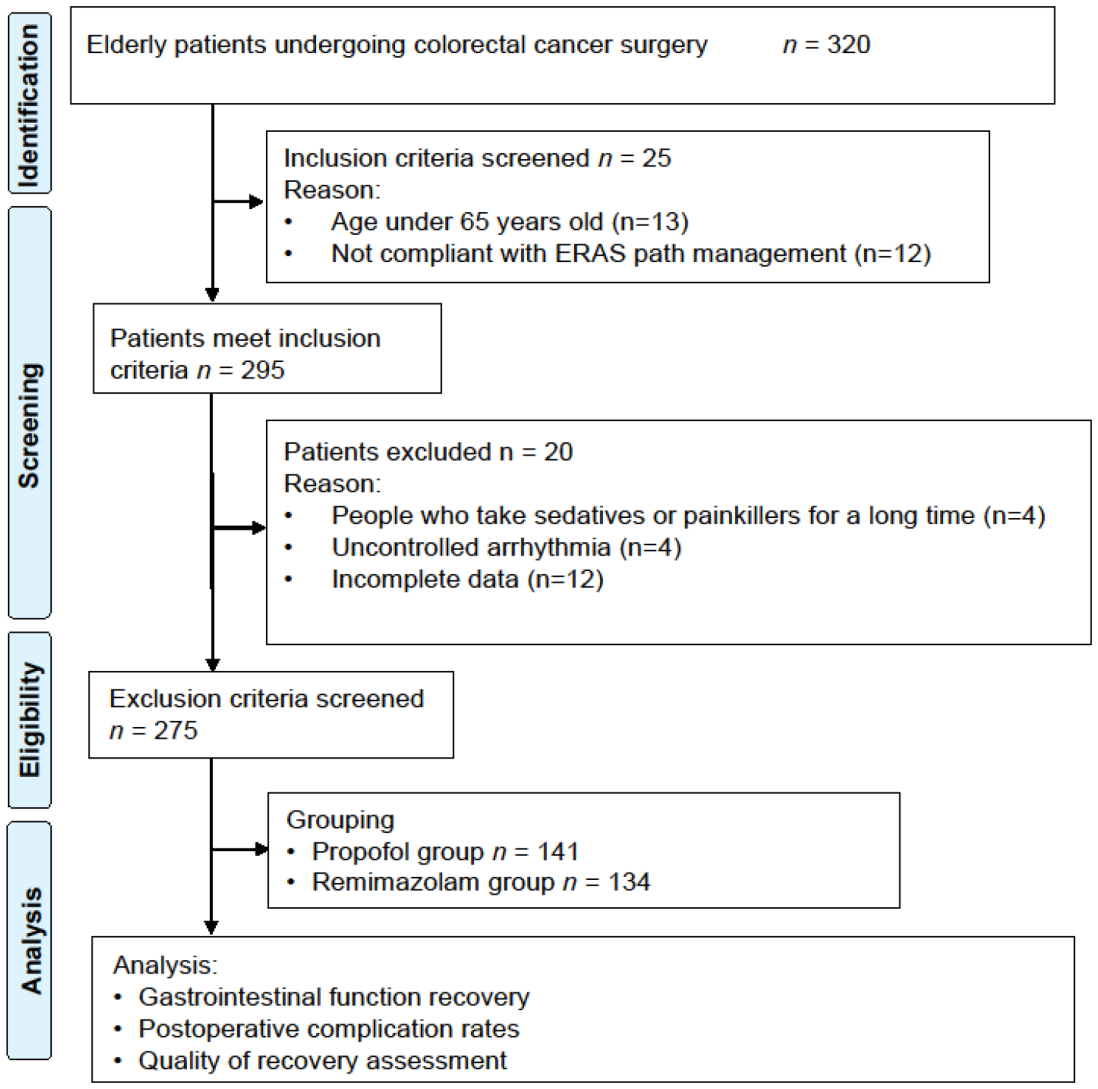
Flowchart of patient recruitment and allocation.

A total of 320 elderly patients undergoing colorectal cancer surgery were initially identified (**Figure 1**). After screening for inclusion criteria, 25 patients were excluded due to age under 65 years (n=13) or non-compliance with ERAS pathway management (n=12), resulting in 295 patients meeting the inclusion criteria. Subsequently, 20 patients were excluded based on exclusion criteria, including long-term use of sedatives or painkillers (n=4), uncontrolled arrhythmia (n=4), and incomplete data (n=12), leaving a final cohort of 275 eligible patients for analysis. These patients were then divided into two groups: the propofol group (n=141) and the remimazolam group (n=134). The primary outcomes assessed included gastrointestinal function recovery, postoperative complication rates, and quality of recovery assessment.

To evaluate the statistical power of the final included sample, we conducted a *post hoc* power analysis using G*Power software (version 3.1.9.7) on the total matched sample. The analysis was based on between-group comparisons of the primary outcome measures, with parameters set as follows: medium effect size (Cohen’s d = 0.5), two-tailed significance level (α = 0.05). The results showed that, with the current sample size, using a two-sided, two-sample t-test with equal variances, the statistical power (1-β) for detecting between-group differences of medium effect size is greater than 95%. This indicates that the matched sample size in this study is sufficient to support meaningful between-group comparisons of the outcomes of interest.

### 2.3 Anesthesia methods

All patients received comprehensive management based on the ERAS concept before surgery. Preoperative education and preparation included detailed explanations of the surgical process, expected outcomes, and postoperative recovery plans to patients and their families, along with psychological support to reduce anxiety and fear. Preoperative nutritional assessments and necessary supplementation were also part of the standard protocol to ensure patients were in optimal condition for surgery. Additionally, all patients fasted for at least 8 hours before surgery and avoided unnecessary bowel preparations to minimize preoperative discomfort and postoperative complications. Smoking cessation and alcohol abstinence were strictly required to reduce the risk of postoperative complications further.

During anesthesia induction, both groups received standardized anesthesia procedures. Patients in the propofol group received an intravenous injection of 1.0 to 2.0 mg/kg of propofol (batch number: H20073642, Yangtze River Pharmaceutical Group, China). Patients in the remimazolam group received an intravenous injection of 0.2 to 0.3 mg/kg of remimazolam over 1 minute (batch number: H20227087, Yichang Renfu Pharmaceutical Co., Ltd., China). The induction dose was selected based on the patient’s body weight. It was fine-tuned in real-time during administration according to the patient’s initial response, hemodynamic changes, and level of consciousness. During anesthesia maintenance, both groups received continuous intravenous infusions. The propofol group received propofol within an allowable titration range of 4.0 to 12.0 mg·kg^-^¹·h^-^¹, while the remimazolam group received remimazolam at 1.0 to 2.0 mg·kg^-^¹·h^-^¹. For elderly patients, the initial maintenance infusion was started at the lower end of the recommended range and was subsequently adjusted according to hemodynamic response, clinical signs of anesthetic depth, and depth-of-anesthesia monitoring. The upper end of the propofol range was not used as a routine target dose but represented the maximum permitted titration limit when clinically required. Both groups also received an infusion of remifentanil (batch number: H20143314, Jiangsu Enhua Pharmaceutical Co., Ltd., China) at a rate of 0.1 to 0.2 μg·kg^-^¹·min^-^¹ to maintain appropriate anesthesia depth and hemodynamic stability. The depth of anesthesia was determined by clinical signs, hemodynamic parameters and anesthesiologist judgment as per the institutional practice. Adequate surgical anesthetic was maintained by modifying the infusion rates of the anesthetic according to the degree of sedation. When hypotension, bradycardia, delayed recovery signs or excessively deep anesthesia were observed, dose reduction was done in elderly patients.

Postoperatively, patients were encouraged to get out of bed as soon as possible and gradually resume eating solid foods according to their tolerance. Pain management and control of nausea and vomiting were key components of ERAS, utilizing multimodal analgesia and antiemetic drugs to reduce patient discomfort and promote rapid recovery. Prevention of PONV was done as per institutional protocol based on individual risk assessment. Prophylaxis consisted of a 5-HT3 receptor antagonist as standard, as well as dexamethasone or other antiemetic medication if clinically indicated. When performed postoperatively, rescue antiemetic treatment was recorded. These comprehensive measures were designed to enhance perioperative management, the quality of the postoperative recovery, and the rate of complications.

ERAS adherence was evaluated based on the standardization of the following components: pre-operative education, avoidance of prolonged fasting, no routine bowel preparation unless clinically indicated, standardized multimodal analgesia, PONV prophylaxis, early mobilization, early oral intake and post-operative nausea and pain control. Adherence for every patient was defined as the percentage of ERAS components that were completed for all the applicable components. Postoperative outcomes were compared between the propofol and remimazolam groups to determine whether there were differences in how the pathway was implemented that might have influenced outcomes.

### 2.4 Outcome measures

The primary outcome measures were the times to postoperative gastrointestinal recovery, including the time to first bowel sound recovery, first flatus passage, first defecation, and first tolerance of solid food.

Secondary outcome measures included changes in serum motilin and gastrin levels at 24 hours postoperatively; the incidence of complications during hospitalization, with a focus on postoperative nausea and vomiting, respiratory depression, hypoxemia, ileus, and postoperative delirium confirmed by twice-daily assessments using the Confusion Assessment Method (CAM); hemodynamic parameters at key time points reflecting intraoperative stability; the Quality of Recovery-15 (QoR-15) score for comprehensive evaluation of patient postoperative experience; and the total length of hospital stay from the day of surgery to discharge. A set of perioperative management variables, such as intraoperative remifentanil dose, postoperative opioid consumption, antiemetic prophylaxis, vasopressor use, fluid administration, and early mobilization, were also tested as potential confounders of gastrointestinal recovery, PONV, delirium, QoR-15 score, and length of hospital stay.

#### 2.4.1 Baseline

The baseline demographic and clinical data of the patients, including gender, age, BMI, and past medical history, were extracted from the initial hospital admission records, nursing assessment records, and previous medical records. The ASA classification was extracted from the preoperative anesthesia visit records (16). The tumor type, size, and TNM stage were confirmed and extracted based on the TNM staging system, using descriptions from archived preoperative imaging reports and postoperative pathology reports (17).

#### 2.4.2 Collection of surgical and anesthesia-related data

The durations of surgery and anesthesia, as well as the total consumption of anesthetic drugs, were extracted from the patients’ electronic anesthesia records. Anesthesiologists routinely completed these records during the perioperative period. Heart rate and mean arterial pressure (MAP) data were extracted from the multi-parameter monitors (model: Philips IntelliVue MX800, manufacturer: Philips Healthcare, country: Netherlands). The numerical values used in the analysis were taken from four fixed perioperative time points (before anesthesia induction, after endotracheal intubation, at the end of surgery, and after extubation). Monitoring and recording at these time points followed our institution’s perioperative monitoring protocol. The length of hospital stay was calculated based on the admission and discharge times recorded in the medical record system. Additional peri-operative variables that may impact post-operative recovery were also collected, such as: surgical approach, type of colorectal resection, intraoperative crystalloid and colloid volumes, estimated blood loss, urine output, vasopressor use, regional anesthesia or nerve block use, total intra-operative opioid exposure and post-operative opioid consumption, antiemetic prophylaxis, time to first post-operative mobilization and compliance with key ERAS pathway components. These variables were obtained from the anesthetic records, surgical notes, nursing records and the ERAS follow-up forms.

#### 2.4.3 Collection of gastrointestinal function-related data

Blood samples were collected before surgery and 24 hours after surgery and immediately sent to the laboratory for processing and analysis. The concentrations of motilin and gastrin were quantified using enzyme-linked immunosorbent assay (ELISA) kits (model: Human Motilin ELISA Kit and Human Gastrin ELISA Kit, manufacturer: Elabscience, country: China). All samples were read and analyzed using an automated microplate reader (model: BioTek Synergy HTX, manufacturer: BioTek Instruments, Inc., country: USA).

The times of first flatus and first bowel movement were recorded by the attending nurses based on patient self-reports. The time of first solid food intake was determined by healthcare professionals based on patient tolerance and recorded in the electronic medical record system when patients actually started eating solid food. The time of bowel sound recovery was assessed using a stethoscope (model: Littmann Classic III Stethoscope, manufacturer: 3M, country: USA). Nurses listened to the patient’s abdomen with a stethoscope and recorded the specific time point when bowel sounds were first heard.

#### 2.4.4 Monitoring and complication recording during hospitalization

During hospitalization, the respiratory rate and oxygen saturation (SpO2) were monitored in real-time using a multi-parameter monitor (model: Philips IntelliVue MX800, manufacturer: Philips Healthcare, country: Netherlands). When the respiratory rate fell below 10 breaths per minute, it was recorded as a respiratory depression event. Oxygen saturation levels below 90% were documented as hypoxemia. The diagnosis of bowel obstruction was based on clinical symptoms and imaging results. Clinical symptoms included abdominal distension, abdominal pain, and cessation of flatus or bowel movements, which were recorded by healthcare professionals based on patient self-reports. Imaging studies used CT scans (model: Siemens SOMATOM Definition Flash, manufacturer: Siemens Healthineers, country: Germany) to confirm the presence of bowel obstruction. Nausea and vomiting were recorded based on patient self-reports and observations by nursing staff. Information on PONV-related perioperative factors was also collected, including antiemetic prophylaxis, rescue antiemetic use, intraoperative remifentanil dose, postoperative opioid consumption, and early oral intake status. These variables were extracted from anesthesia records, medication administration records, nursing records, and ERAS follow-up forms.

#### 2.4.5 Assessment and recording of delirium

The incidence and duration of delirium within the first seven days after surgery were recorded. The incidence of delirium was assessed using the Confusion Assessment Method (CAM). Yantaishan Hospital has a routine CAM screening process for elderly surgical patients. Patients were evaluated daily from postoperative day one to day seven by healthcare professionals who were trained uniformly. The assessment included four core features: acute onset and fluctuating course, inattention, disorganized thinking, and altered level of consciousness. If a patient met the CAM criteria, the event was recorded as a delirium incident and entered into the electronic medical record system. Delirium assessments were conducted twice daily, once from 8 AM to 10 AM and again from 6 PM to 8 PM. Possible delirium-causing clinical information was also obtained, if available, such as a baseline cognitive status or documented cognitive impairment, frailty-related clinical data, exposure to anticholinergic medication perioperatively, opioid use postoperatively, admission to the intensive care unit (ICU), postoperative infection, postoperative hypoxemia, and other postoperative complications. In additional adjusted analyses, these factors were taken into account to help reduce the possibility of confounding outcomes of postoperative delirium.

#### 2.4.6 Assessment of recovery quality

Recovery quality was assessed using the Quality of Recovery-15 (QoR-15) scale. QoR-15 scores were extracted from the patient self-assessment questionnaires archived as part of Yantaishan Hospital’s ERAS protocol, which were completed on postoperative day 7. These questionnaires are a routine component of postoperative follow-up for patients, which evaluate five domains: physical comfort, psychological support, emotional state, physical independence, and pain (18). The scale comprised 15 items, each scored on a scale from 0 to 10, with a maximum total score of 150. Higher scores indicated better recovery quality. The QoR-15 scale typically categorizes total scores into four intervals: 0–89 indicates poor recovery, 90–121 indicates moderate recovery, 122–135 indicates good recovery, and 136–150 indicates excellent recovery.

### 2.5 Data collection

Data were collected by research nurses or assistants who were uniformly trained and blinded to the group assignments. Baseline data were collected during preoperative visits. Surgical and anesthesia data were recorded in real-time from the monitoring devices’ data acquisition systems and anesthesia records. Gastrointestinal recovery times were documented through three daily scheduled interviews with patients and physical examinations. Complications were recorded through daily rounds and medical record reviews. Blood samples were collected at designated time points and immediately sent for testing. The QoR-15 questionnaire was completed by patients on postoperative day 7, with researchers providing necessary explanations but no leading guidance. All data were directly entered into electronic case report forms, with logical checks set up to minimize errors. The study will strictly adhere to the protocol, and any cases with incomplete data will be excluded from the final analysis.

### 2.6 Data analysis

The data were analyzed in SPSS V.28.0. Normality of continuous variables was determined by the Shapiro–Wilk test and presented as mean ± SD or as median (interquartile range) as appropriate. Independent-samples t-tests were used for normally distributed variables, and Mann–Whitney U tests were used for those that were not normally distributed for between-group comparisons.

The results for categorical variables are reported as frequencies and percentages. Chi-square tests were performed where the expected cell counts were not too small, and Fisher’s exact test was performed for sparse categorical data with expected cell counts less than 5.

Propensity score matching was used with a multivariable logistic regression model of age, sex, BMI, smoking status, drinking history, ASA classification, tumor location, tumor size, TNM stage, hypertension, diabetes mellitus and coronary heart disease, to minimize the baseline imbalance. Standard deviations of the logit of the propensity score were used as a caliper for nearest-neighbor matching without replacement. After matching, a total of 128 patients with propofol and 115 with remimazolam were included in the study.

Standardized mean differences were used to evaluate covariate balance, where values <0.10 are considered acceptable. Given that multiple postoperative outcomes were examined, secondary findings were interpreted with caution, focusing on effect estimates, 95% confidence intervals, consistency across related outcomes, and clinical relevance. A two-sided P value <0.05 was considered statistically significant.

## 3 Results

### 3.1 Baseline data before propensity score matching

In the baseline characteristics comparison before propensity score matching, significant differences were observed in age, anesthesia classification, and tumor size between the Propofol group and the Remimazolam group (**Table 1**). Patients in the propofol group were significantly older than those in the remimazolam group (P<0.001), which may lead to different tolerances to anesthesia and surgical stress. There was also a significant difference in the distribution of ASA classifications (P = 0.037); compared with the remimazolam group, more patients in the propofol group were classified as ASA I, suggesting that their baseline health status might be better. Additionally, the tumor size in the remimazolam group was significantly larger than that in the propofol group (P = 0.003), which may indicate potential differences in the extent or complexity of surgery. For other parameters, no significant differences were noted. Gender distribution, BMI, smoking status, drinking habits, types of tumors, TNM stage, hypertension, diabetes mellitus, and coronary heart disease showed no significant differences between the two groups (all P>0.05).

**Table 1:** Baseline characteristics comparison before propensity score matching.

Measure	Propofol group (n=141)	Remimazolam group (n=134)	t/χ2	P
Gender			0.973	0.324
Female	59 (41.84%)	64 (47.76%)		
Male	82 (58.16%)	70 (52.24%)		
Age	78.11 ± 4.95	75.36 ± 4.28	4.928	< 0.001
BMI	22.04 ± 3.03	21.82 ± 3.45	0.554	0.580
Smoker	25 (17.73%)	20 (14.93%)	0.395	0.530
Drinking	24 (17.02%)	18 (13.43%)	0.684	0.408
Anesthesia Classification			4.358	0.037
ASA I	34 (24.11%)	19 (14.18%)		
ASA II	107 (75.89%)	115 (85.82%)		
Tumor Types			1.176	0.555
Right Colon Tumor	97 (68.79%)	88 (65.67%)		
Left Colon Tumor	30 (21.28%)	27 (20.15%)		
Rectal Tumor	14 (9.93%)	19 (14.18%)		
Tumor size (mm)	44.12 ± 5.82	46.19 ± 5.68	2.981	0.003
TNM stage			1.480	0.477
I	56 (39.72%)	44 (32.84%)		
II	55 (39.01%)	60 (44.78%)		
III	30 (21.28%)	30 (22.39%)		
Hypertension	45 (31.91%)	38 (28.36%)	0.412	0.521
Diabetes mellitus	27 (19.15%)	24 (17.91%)	0.070	0.792
Coronary heart disease	15 (10.64%)	8 (5.97%)	1.954	0.162

BMI, body mass index; ASA, American Society of Anesthesiologists; TNM, Tumor, Node, Metastasis.

### 3.2 Baseline data after propensity score matching

After variable-ratio propensity score matching, 128 patients in the propofol group and 115 patients in the remimazolam group were retained for analysis. The unequal group sizes resulted from the variable-ratio matching strategy and exclusion of patients outside the acceptable caliper range. Baseline balance was assessed primarily using standardized mean differences rather than P-values (**Table 2**). Gender distribution, age, BMI, smoking status, drinking habits, anesthesia classification, tumor types, tumor size, TNM stage, hypertension, diabetes mellitus, and coronary heart disease all indicated no significant differences between the two groups (all P>0.05). This confirms that the propensity score matching effectively balanced the baseline characteristics, ensuring comparability between the two groups of patients. This is crucial for accurately assessing the effects of the anesthetic drugs themselves, as it maximizes control over bias caused by baseline confounding factors. The distribution of standardized mean differences before and after matching is presented in **Supplementary Figure 1**, demonstrating improved covariate balance after matching.

**Table 2:** Baseline characteristics comparison after propensity score matching.

Measure	Propofol group (n=128)	Remimazolam group (n=115)	t/χ²	P	SMD
Gender			0.115	0.734	0.044
Female	54 (42.19%)	51 (44.35%)			
Male	74 (57.81%)	64 (55.65%)			
Age	74.96 ± 4.63	75.12 ± 3.99	0.287	0.774	0.037
BMI	21.87 ± 2.97	22.15 ± 3.26	0.718	0.473	0.09
Smoker	20 (15.62%)	14 (12.17%)	0.6	0.439	0.1
Drinking	16 (12.50%)	12 (10.43%)	0.253	0.615	0.065
Anesthesia Classification			0.232	0.63	0.062
ASA I	23 (17.97%)	18 (15.65%)			
ASA II	105 (82.03%)	97 (84.35%)			
Tumor Types			0.9	0.637	0.121
Right Colon Tumor	92 (71.88%)	79 (68.70%)			
Left Colon Tumor	27 (21.09%)	24 (20.87%)			
Rectal Tumor	9 (7.03%)	12 (10.43%)			
Tumor size (mm)	45.22 ± 5.56	44.69 ± 5.39	0.757	0.45	0.097
TNM stage			1.081	0.583	0.132
I	55 (42.97%)	42 (36.52%)			
II	49 (38.28%)	48 (41.74%)			
III	24 (18.75%)	25 (21.74%)			
Hypertension	38 (29.69%)	34 (29.57%)	0	0.983	0.003
Diabetes mellitus	25 (19.53%)	19 (16.52%)	0.37	0.543	0.078
Coronary heart disease	12 (9.38%)	6 (5.22%)	1.527	0.217	0.16

BMI, body mass index; ASA, American Society of Anesthesiologists; TNM, Tumor, Node, Metastasis; SMD, standardized mean difference.

### 3.3 Surgical and anesthesia-related data

In the comparison of surgical and anesthesia-related parameters between the Propofol group and the Remimazolam group, several significant differences were noted (**Table 3**). The heart rate at the end of surgery was significantly lower in the Propofol group compared to the Remimazolam group (P = 0.019), indicating a difference in cardiovascular response during the surgical procedure. Additionally, MAP at the end of surgery was also significantly lower in the Propofol group than in the Remimazolam group (P = 0.022), suggesting a differential hemodynamic effect between the two anesthetic agents. Statistically, the length of hospital stay was shorter with the remimazolam group than the propofol group, with an absolute mean difference of 0.47 days (95% CI 0.13 to 0.81; 11.92 ± 1.13 days remimazolam group vs 12.39 ± 1.52 days propofol group; P = 0.007). While this difference is statistically significant, it was a relatively small decrease in the length of stay in the hospital. But there were no significant differences in other parameters (all P>0.05). The maintenance range was reported, as this was the range in which the propofol dose could be titrated and not the dose routinely administered. The total propofol consumption and the total duration of anesthesia were used to calculate the estimated dose-adjusted exposure to propofol as about 5.14 mg/kg/h for a mean body weight of 60 kg. This means the average dose was towards the lower to middle end of the reported titration range and not at the top end. Patient-level body weight data should be used in the interpretation of final dose-exposure values. A list of other perioperative variables that might affect postoperative recovery is given in **Supplementary Table 1**. Comparisons were made between the groups for surgical approach, type of resection, intraoperative fluid management, estimated blood loss, vasopressor use, regional anesthesia/nerve block use, postoperative opioid use, antiemetic prophylaxis, early mobilization, and adherence to overall ERAS protocol. To better define potential perioperative confounding factors, these variables were added.

**Table 3:** Surgical and anesthesia-related parameters comparison.

Measure	Propofol group (n=128)	Remimazolam group (n=115)	t	P
Surgical Time (minutes)	224.91 ± 28.39	222.17 ± 27.54	0.763	0.446
Anesthesia Duration (minutes)	247.84 ± 30.72	246.20 ± 31.16	0.413	0.68
Propofol Consumption	1273.08 ± 109.03	—	—	—
Propofol cumulative dose, mg/kg*	21.22 ± 1.82	—	—	—
Propofol duration-adjusted dose, mg/kg/h*	5.14 ± 0.78	—	—	—
Remimazolam Consumption	—	263.45 ± 19.12	—	—
Remimazolam cumulative dose, mg/kg*	—	4.39 ± 0.32	—	—
Remimazolam duration-adjusted dose, mg/kg/h*	—	1.07 ± 0.16	—	—
Remifentanil Dose (mg)	2.32 ± 0.23	2.29 ± 0.21	1.112	0.267
Heart Rate Before Induction	78.02 ± 5.54	77.25 ± 5.97	1.053	0.294
Heart Rate After Intubation	75.82 ± 8.12	74.19 ± 7.68	1.601	0.111
Heart Rate At the End of Surgery	74.47 ± 6.76	76.32 ± 5.44	2.362	0.019
Heart Rate After Extubation	79.14 ± 6.87	77.83 ± 5.35	1.67	0.096
MAP Before Induction, mmHg	96.58 ± 7.91	97.24 ± 8.35	0.64	0.523
MAP After Intubation, mmHg	83.72 ± 6.51	85.13 ± 5.49	1.822	0.07
MAP At the End of Surgery, mmHg	88.33 ± 6.11	90.58 ± 8.74	2.303	0.022
MAP After Extubation, mmHg	100.50 ± 8.14	98.84 ± 7.25	1.676	0.095
Length of Hospital Stay, days	12.39 ± 1.52	11.92 ± 1.13	2.746	0.007

MAP, Mean Arterial Pressure.

### 3.4 Gastrointestinal function recovery after propensity score matching

**Figure 2:**
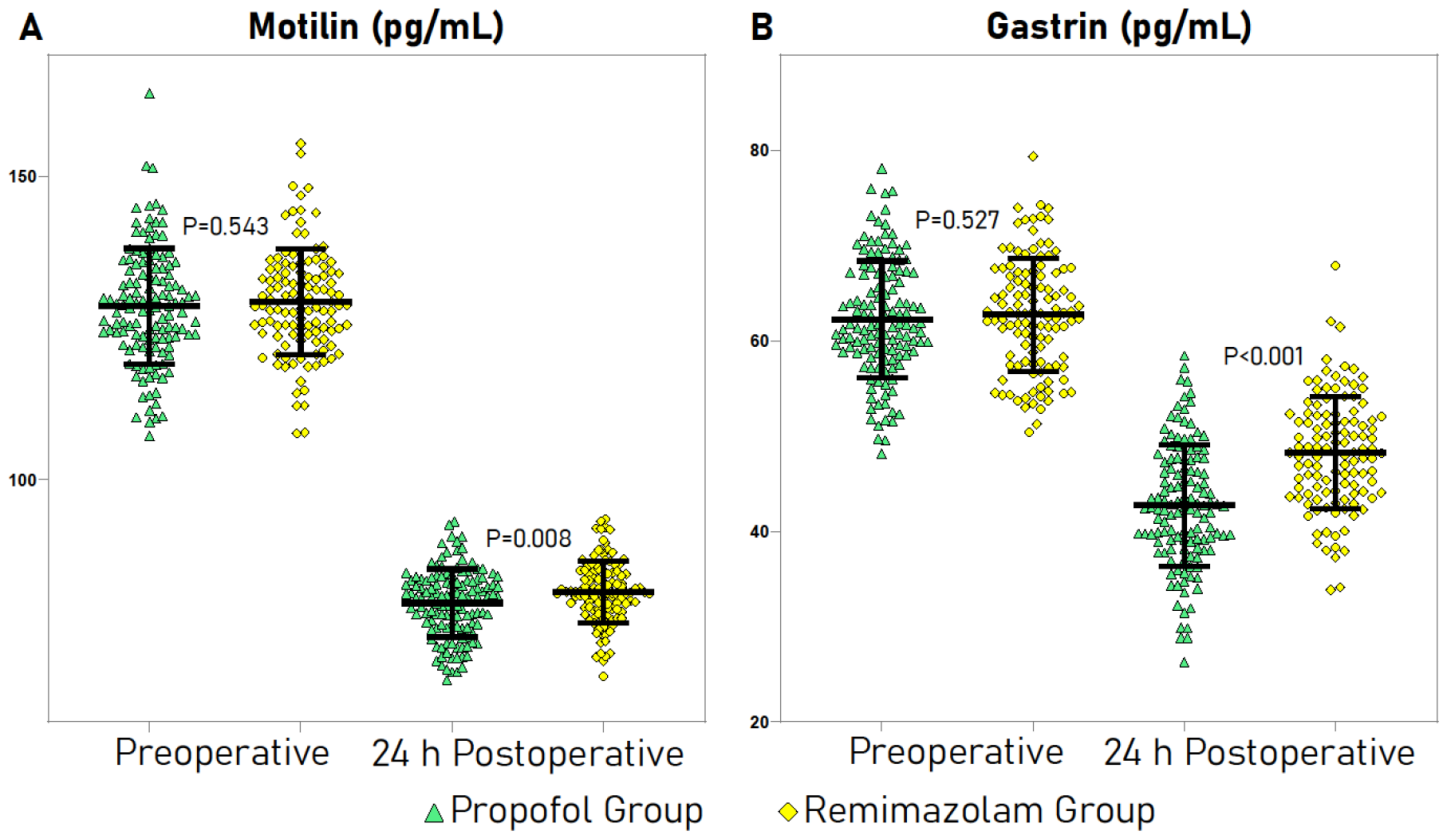
Comparison of Motilin and Gastrin Levels Before and After Surgery. **(A)** Motilin (pg/mL); **(B)** Gastrin (pg/mL).

For motilin, there was no significant difference between the two groups preoperatively (P = 0.543; **Figure 2**). However, 24 hours postoperatively, motilin levels were significantly lower in the Propofol group compared to the Remimazolam group (P = 0.008), indicating a differential effect on motilin levels following surgery depending on the anesthetic used. Regarding gastrin, no significant difference was found between the two groups preoperatively (P = 0.527). In contrast, 24 hours postoperatively, gastrin levels were significantly lower in the Propofol group than in the Remimazolam group (P<0.001), suggesting that the Propofol group experienced a greater reduction in gastrin levels after surgery. These findings indicate an association between anesthetic group and postoperative motilin and gastrin levels at 24 hours; however, because hormone levels were measured at only one postoperative time point, these results should be considered exploratory.

The time to first passage of flatus was shorter in the remimazolam group, with a mean difference of 0.74 h (95% CI: 0.25 to 1.23; P = 0.003). The corresponding mean differences were 1.48 h for first bowel movement (95% CI: 0.54 to 2.42; P = 0.002), 1.89 h for first solid food intake (95% CI: 0.49 to 3.29; P = 0.009), and 0.84 h for bowel sound recovery (95% CI: 0.18 to 1.50; P = 0.013) (**Table 4**). These findings collectively suggest that patients in the remimazolam group experienced statistically shorter gastrointestinal recovery times than those in the propofol group, although the absolute differences were modest.

**Table 4:** Postoperative Gastrointestinal Function Recovery Times.

Measure	Propofol group (n=128)	Remimazolam group (n=115)	Mean difference	95% CI	t	P
First Passage of Flatus (h)	20.25 ± 1.98	19.51 ± 1.86	0.74 h	0.25 to 1.23	2.991	0.003
First Bowel Movement (h)	48.25 ± 3.82	46.77 ± 3.64	1.48 h	0.54 to 2.42	3.074	0.002
Time to First Solid Food Intake (h)	83.61 ± 5.83	81.72 ± 5.26	1.89 h	0.49 to 3.29	2.635	0.009
Time to Bowel Sound Recovery (h)	25.38 ± 2.57	24.54 ± 2.66	0.84 h	0.18 to 1.50	2.501	0.013

### 3.5 Postoperative complications incidence

The incidence of nausea and vomiting was significantly higher in the Propofol group compared to the Remimazolam group (P = 0.032), indicating a greater likelihood of experiencing these symptoms in patients receiving propofol (**Table 5**). Additionally, the total number of complications was also significantly higher in the Propofol group than in the Remimazolam group (P = 0.007), suggesting an overall increased risk of adverse events in this group. For other complications, no significant differences were noted. The rates of respiratory depression (P = 0.687), intestinal obstruction (P = 0.354), and hypoxemia (P = 0.691) were similar between the two groups, indicating that these specific complications did not differ significantly based on the anesthetic agent used.

**Table 5:** Comparison of postoperative complications.

Measure	Propofol group (n=128)	Remimazolam group (n=115)	OR for remimazolam vs propofol	95% CI	χ²	P
Respiratory Depression	7 (5.47%)	5 (4.35%)	0.79	0.24 to 2.55	0.162	0.687
Intestinal Obstruction	6 (4.69%)	2 (1.74%)	0.36	0.07 to 1.82	0.858	0.354
Hypoxemia	3 (2.34%)	1 (0.87%)	0.37	0.04 to 3.56	0.157	0.691
Nausea and Vomiting	17 (13.28%)	6 (5.22%)	0.36	0.14 to 0.95	4.597	0.032
Total	33 (25.78%)	14 (12.17%)	0.4	0.20 to 0.79	7.19	0.007

**Figure 3:**
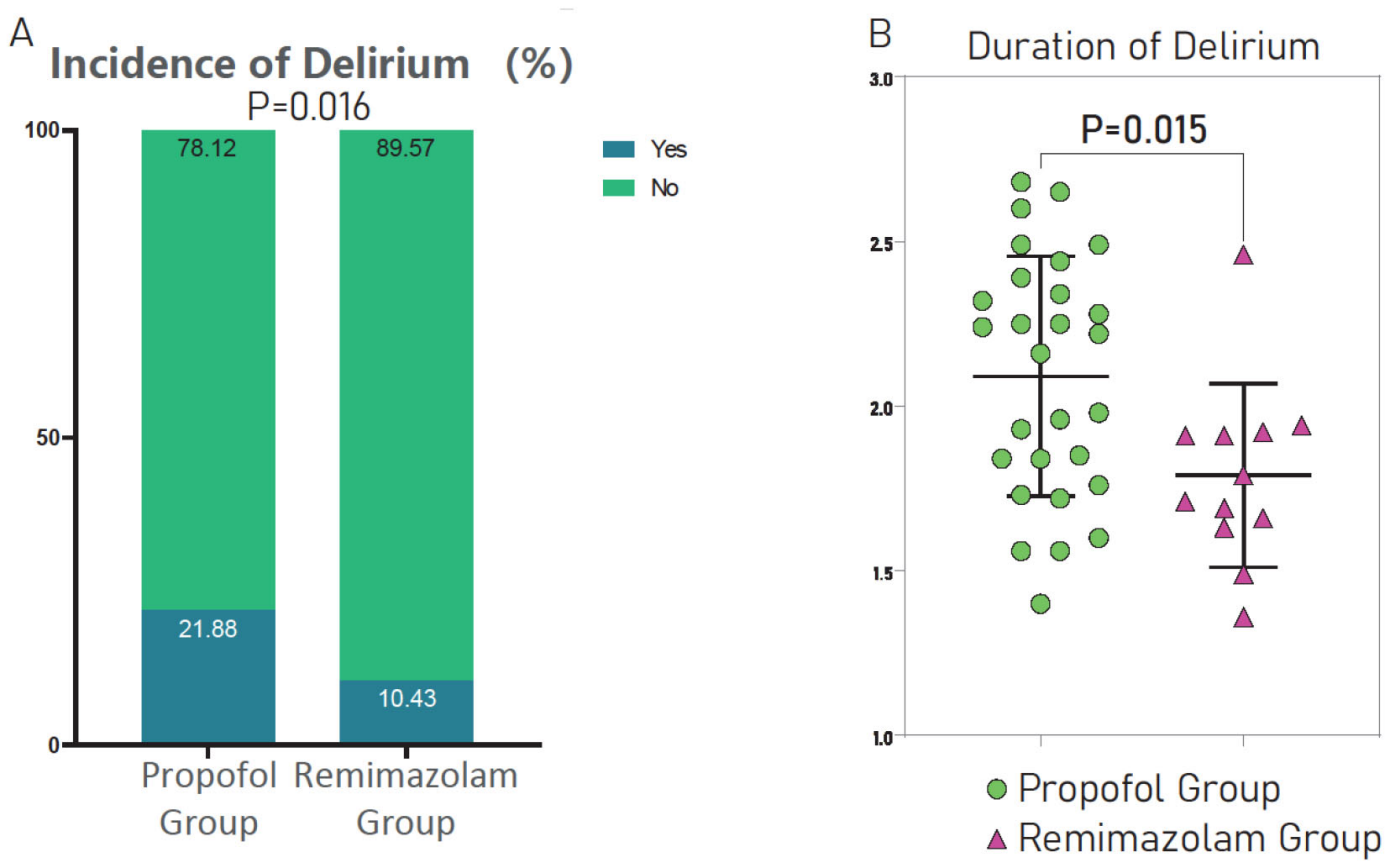
Incidence and duration of postoperative delirium. **(A)** Incidence of Delirium (%); **(B)** Duration of Delirium.

The incidence of delirium was significantly higher in the Propofol group compared to the Remimazolam group (P = 0.016), indicating a greater likelihood of developing delirium in patients receiving propofol (**Figure 3**). Additionally, the duration of delirium was also significantly longer in the Propofol group than in the Remimazolam group (P = 0.015), suggesting that not only is delirium more common, but it also persists for a longer period in these patients. This association should be interpreted cautiously because postoperative delirium is multifactorial and may be influenced by baseline vulnerability and perioperative factors. Additional adjusted logistic regression analyses for PONV and postoperative delirium are presented in **Supplementary Table 2**. These analyses were performed to further account for clinically relevant perioperative confounders, including opioid exposure, antiemetic prophylaxis, ICU admission, infection, hypoxemia, and other postoperative complications.

### 3.6 Postoperative recovery quality

The QoR-15 score was significantly higher in the Remimazolam group compared to the Propofol group (P<0.001), indicating better overall quality of recovery in patients receiving remimazolam (**Table 6**). Regarding recovery grades, the distribution also differed significantly between the two groups (P = 0.006). The Remimazolam group had a higher proportion of patients with a “Good” recovery grade and a lower proportion with a “Moderate” recovery grade compared to the Propofol group. This suggests that patients in the Remimazolam group experienced better recovery outcomes as assessed by both quantitative scores and qualitative grades.

**Table 6:** Comparison of QoR-15 scores and recovery grades.

Measure	Propofol group (n=128)	Remimazolam group (n=115)	t/χ2	P
QoR-15 score	119.89 ± 12.82	125.44 ± 9.95	3.790	< 0.001
Grade			10.222	0.006
Moderate	73 (57.03%)	42 (36.52%)		
Good	43 (33.59%)	57 (49.57%)		
Excellent	12 (9.38%)	16 (13.91%)		

### 3.7 Effect sizes

The analysis of effect sizes (Cohen’s d) between the remimazolam and propofol groups for elderly patients undergoing colorectal cancer surgery reveals nuanced differences across various perioperative parameters (**Table 7**). For demographic and tumor characteristics, negligible differences were observed with Cohen’s d values close to zero for age (-0.037), BMI (-0.092), tumor size (0.097), surgical time (0.098), and anesthesia duration (0.053), indicating minimal variance attributable to anesthetic choice. The consumption ratio of remimazolam to propofol was notably high (12.584). Regarding intraoperative parameters, minor differences were found in heart rate changes at different stages and MAP, with more pronounced effects post-extubation for heart rate (0.212) and at the end of surgery for MAP (-0.301). Postoperative recovery parameters such as length of hospital stay (0.347), motilin levels post-surgery (-0.344), gastrin levels post-surgery (-0.896), times to first passage of flatus (0.384), first bowel movement (0.395), first solid food intake (0.339), and bowel sound recovery (0.321) showed moderate effect sizes, suggesting better outcomes in the remimazolam group. Additionally, the QoR-15 score indicated a higher quality of recovery in the remimazolam group (-0.393), further supporting its potential benefits over propofol in this patient population.

**Table 7:** Effect sizes of perioperative parameters between remimazolam and propofol groups in elderly patients undergoing colorectal cancer surgery.

Measure	Cohen d
Age	-0.037
BMI	-0.092
Tumor size (mm)	0.097
Surgical Time (minutes)	0.098
Anesthesia Duration	0.053
Remimazolam Consumption/Propofol Consumption	12.584
Remifentanil Dose (mg)	0.143
Heart Rate Before Induction	0.135
Heart Rate After Intubation	0.206
Heart Rate At the End of Surgery	-0.300
Heart Rate After Extubation	0.212
MAP (mmHg) Before Induction	-0.082
MAP (mmHg) After Intubation	-0.234
MAP (mmHg) At the End of Surgery	-0.301
MAP (mmHg) After Extubation	0.215
Length of Hospital Stay	0.347
Motilin (pg/mL) pre-surgery	-0.078
Motilin (pg/mL) post-surgery	-0.344
Gastrin (pg/mL) pre-surgery	-0.081
Gastrin (pg/mL) post-surgery	-0.896
First Passage of Flatus (h)	0.384
First Bowel Movement (h)	0.395
Time to First Solid Food Intake (h)	0.339
Time to Bowel Sound Recovery (h)	0.321
QoR-15 score	-0.393

## 4 Discussion

The study examined how remimazolam anesthesia affected elderly patients undergoing radical cancer resection within the ERAS framework. Results showed that, compared with propofol, remimazolam was associated with higher postoperative recovery quality scores, shorter gastrointestinal recovery times, and lower observed rates of certain complications. These findings suggest that remimazolam may be a potentially useful anesthetic option for this specific and vulnerable patient group; however, causal interpretation is limited by the observational cohort design.

One key finding was that remimazolam demonstrated more favorable hemodynamic characteristics at the end of surgery. Researchers observed decreases in heart rate and mean arterial pressure in the propofol group at the end of surgery, confirming propofol’s known dose-dependent cardiovascular suppression (19). In contrast, remimazolam provided better hemodynamic stability. For elderly patients, who often have reduced cardiovascular reserves and lower tolerance for blood pressure fluctuations, this stability is crucial (20). Unstable hemodynamics can affect the perfusion of vital organs like the intestines, potentially leading to postoperative ileus (21). Therefore, remimazolam not only enhanced intraoperative safety but also created a more stable physiological environment for postoperative recovery, which likely contributed to shorter hospital stays. Importantly, it should be noted that the range of propofol maintenance reported should not be interpreted as the dose routinely administered to all patients. The dosage of Propofol was adjusted on a case-by-case basis, especially in the elderly, based on the degree of anesthesia and hemodynamic response. However, as exposure to propofol can affect hemodynamic stability, recovery profile, PONV and delirium, we have provided additional dose-exposure variables to improve transparency and note that this is an important factor to consider when interpreting between-group differences.

Statistically shorter recovery times in the gastrointestinal field with remimazolam. But the absolute inter-group differences have been small, only 0.74 h for first flatus and 1.89 h for first solid food intake. Thus, it should be understood that these results reflect small improvements in the early markers of gastrointestinal recovery but not as large individual clinical effects. This clinical observation was backed up by gastrointestinal hormone analysis. Motilin and gastrin levels were increased in the remimazolam group at 24 hours post-surgery. These findings might be a result of hormonal postoperative neuroendocrine differences between groups and not of the direct gastrointestinal recovery-promoting effect of remimazolam. Motilin is important in the stimulation of gastric emptying and the initiation of migrating motor complexes, and gastrin has effects on gastric acid secretion and gastric mucosal growth (22, 23). The increase in motilin after surgery may directly stimulate gastrointestinal motility, and gastrin may aid in the maintenance of gastrointestinal function and the mucosal barrier. The results of these biomarkers can lead to the development of hypotheses about post-surgical gastrointestinal recovery, and the current study was not designed to test a causal hormonal pathway. Propofol has been proposed to act as an inhibitor of neuroendocrine response, or directly on gastrointestinal smooth muscle and therefore decrease the release of these hormones (24). We found that anesthetic choice may be related to changes in postoperative gastrointestinal hormone levels, based on the limited sampling schedule; no mechanistic inferences could be made. It is important to note that even small enhancements in gastrointestinal recovery can be relevant when combined with other signs of recovery, such as earlier initiation of enteral feeding, lesser patient discomfort and a slight decrease in hospital stay in the context of a multimodal ERAS pathway.

Moreover, the remimazolam group performed better regarding complications. The occurrence of PONV was notably lower, which was a clear advantage. PONV is a common and distressing complication that can delay oral intake, ambulation, and discharge. Although the causes of PONV are complex, the choice of anesthetic is a recognized factor (25). While propofol has some antiemetic effects at low doses, it tends to be associated with PONV when used as the main maintenance agent, possibly due to its mechanism of action or interactions with other perioperative drugs (26). We observed that remimazolam reduced PONV, consistent with its pharmacological profile as a benzodiazepine (27). Additionally, although opioids such as remifentanil are known risk factors for PONV, the remifentanil doses used were comparable between the two groups in this study, which further supports that the observed differences in PONV incidence are primarily attributable to the different characteristics of the two sedative drugs themselves. Benzodiazepines generally do not cause vomiting. Thus, remimazolam not only improved patient comfort but also enhanced their recovery process. Opioid exposure, the use of antiemetic prophylaxis, fluid therapy, mobilization and adherence to ERAS may also affect PONV and gastrointestinal recovery, and the results should be viewed with caution. To account for potential confounding, variables that were available in the perioperative period were added to the analyses. However, it is possible that something that we did not account for could have contributed to the association observed. Regarding other complications such as respiratory depression and bowel obstruction, there were no differences between the two groups. This may be due to the overall low incidence of these events in patients following the ERAS pathway, and the study’s sample size may have limited power to detect such low-frequency event differences between groups.

The two groups also showed differences in neurocognitive outcomes. The remimazolam group had a lower incidence of postoperative delirium and shorter duration of delirium, which held profound clinical implications. Postoperative delirium is a major issue for elderly surgical patients, often linked to higher morbidity, prolonged hospital stays, and long-term cognitive decline (28). Propofol provides sedation by acting on GABA-A receptors. Still, this action could disrupt cholinergic neurotransmission and neuronal circuit integrity, increasing the risk of delirium, especially with prolonged or high-dose use (29, 30). In contrast, remimazolam, although also acting on GABA-A receptors, had a very short half-life due to its unique metabolism through tissue esterases, independent of organ function (31). This led to cleaner and more predictable recovery with minimal residual drug effects. This rapid and complete clearance likely reduced the burden contributing to delirium, a hypothesis supported by our findings. While previous studies warned against benzodiazepines in intensive care settings due to their potential to increase delirium risk, our results suggested that remimazolam’s unique pharmacokinetics might change this perspective, particularly in surgical settings (32).

These benefits, such as improved hemodynamics, faster gastrointestinal function recovery, and fewer complications, were evident in the objective assessment of recovery quality. The remimazolam group had higher QoR-15 scores and more favorable recovery grade distributions, providing comprehensive validation of its efficacy. The QoR-15 scale covers various aspects, including physical comfort, mental health, and physical independence, indicating that remimazolam’s advantages extended beyond physiological parameters to encompass overall patient experience. Better recovery quality meant patients could return to normal activities more quickly and experienced fewer adverse effects, which was particularly important for elderly patients with limited physiological reserves and higher complication risks (33).

These findings offer observation evidence that could be useful in guiding future clinical research and decisions in the perioperative setting. Remimazolam can be considered as an alternative sedative in ERAS anesthesia in elderly colorectal surgery patients, especially in patients with hemodynamic instability or a high risk of delirium after surgery. But caution should be used in interpreting this due to a lack of randomization for treatment allocation. Results of this study can also be applied to the future update of the ERAS guidelines with respect to the choice of anesthetic drugs, taking into account the elderly population.

However, there are some limitations to consider. First, this study was a single-center prospective cohort study and not a randomized controlled trial; thus, despite propensity score matching, selection bias, residual confounding, and unmeasured confounding cannot be ruled out. Second, there were a number of peri-operative factors that might have influenced the results for postoperative recovery, such as surgical technique, type of resection, fluid management, use of vasopressors, regional anesthesia, opioid consumption, anti-emetic prophylaxis, timing of mobilization and adherence to ERAS. While the variables available were gathered and compared, there is a possibility that some factors may not have been fully captured. Third, multiple postoperative outcomes were examined, and the findings of those outcomes should be interpreted as exploratory, as this could lead to a Type I error. Fourth, motilin and gastrin were only tested preoperatively and 24 hours post-surgery, and the results of these hormones, thus, should be viewed as exploratory and not a mechanistic pathway. Lastly, follow-up only included the hospitalization period, so long-term effects like cognitive dysfunction, cancer recurrence, or long survival could not be assessed. Thus, multicenter, randomized trials with longer follow-up are warranted to confirm those results.

Future research needs to address the aforementioned limitations. Large, multicenter, prospective randomized controlled trials should be conducted, with postoperative gastrointestinal function recovery, complication rates, and quality of recovery as primary outcome measures, to provide higher-level evidence. In addition, long-term follow-up should be included to evaluate the impact of anesthetic choices on tumor recurrence and sustained cognitive function. Furthermore, future mechanistic studies should include serial monitoring of perioperative inflammatory markers and gastrointestinal hormone dynamics to determine whether changes in motilin, gastrin, or other biomarkers are directly involved in postoperative gastrointestinal recovery. Finally, on the basis of confirming its clinical advantages, conducting a pharmacoeconomic evaluation to assess its cost-effectiveness comprehensively will be of great significance for the reasonable positioning and promotion of this drug within the healthcare system.

## 5 Conclusion

In conclusion, remimazolam anesthesia compared to propofol was linked to more stable intraoperative hemodynamic parameters, shorter gastrointestinal recovery times, reduced observed incidences of PONV and delirium, and increased QoR-15 scores in elderly patients who underwent colorectal cancer surgery in the context of an ERAS. However, because the cohort design of these studies was non-randomized, the absolute differences in gastrointestinal recovery were small, and the possibility of residual confounding exists, the results must be interpreted with caution and as associations rather than causal effects. The hormone differences that were observed may also be considered exploratory. The results of these multicenter, randomized trials with long-term follow-up are necessary to validate these results and to determine their clinical implications.

## Data Availability

The original contributions presented in the study are included in the article/**Supplementary Material**. Further inquiries can be directed to the corresponding author/s.

## Supplementary material

Supplementary Figure 1 Covariate balance before and after propensity score matching.

Supplementary Table 1 Perioperative Management Variables and ERAS Adherence After Propensity Score Matching.

Supplementary Table 2 Association of Remimazolam with PONV and Postoperative Delirium After Propensity Score Matching.
