# Supplementary figures and images for: Application of remimazolam anesthesia in elderly patients undergoing radical resection for colorectal cancer: a cohort study on gastrointestinal recovery and complication rates
Source: Front Oncol. 2026 Jul 8;16:1867238. doi: 10.3389/fonc.2026.1867238 (PMC13388177; doi:10.3389/fonc.2026.1867238)

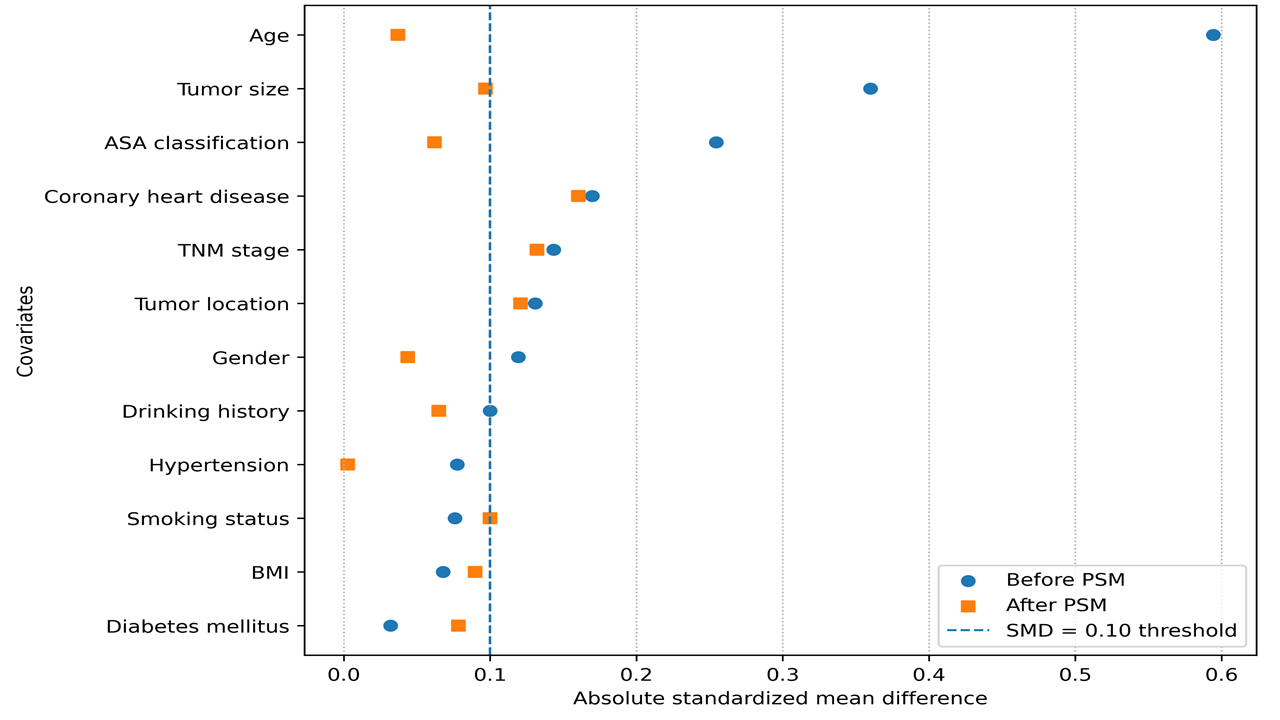

Supplement: Supplementary Figure 1 — Covariate balance before and after propensity score matching. [file Image1.tif]
