# Supplementary material for: Application of remimazolam anesthesia in elderly patients undergoing radical resection for colorectal cancer: a cohort study on gastrointestinal recovery and complication rates
Source: Front Oncol. 2026 Jul 8;16:1867238. doi: 10.3389/fonc.2026.1867238 (PMC13388177; doi:10.3389/fonc.2026.1867238)
Supplement: Supplementary Table 1 — Perioperative Management Variables and ERAS Adherence After Propensity Score Matching. [file Table1.docx]

**Supplementary Table S1. Perioperative Management Variables and ERAS Adherence After Propensity Score Matching**

| **Variable** | **Propofol Group (n=128)** | **Remimazolam Group (n=115)** | **P value** |
| --- | --- | --- | --- |
| Laparoscopic surgery, n (%) | 128 (100.00%) | 115 (100.00%) | — |
| Open surgery, n (%) | 0 (0.00%) | 0 (0.00%) | — |
| Right colectomy, n (%) | 92 (71.88%) | 79 (68.70%) | 0.637 |
| Left colectomy, n (%) | 27 (21.09%) | 24 (20.87%) | |
| Rectal resection, n (%) | 9 (7.03%) | 12 (10.43%) | |
| Intraoperative crystalloid volume, mL | 1850 ± 310 | 1815 ± 295 | 0.368 |
| Intraoperative colloid volume, mL | 210 ± 96 | 198 ± 90 | 0.316 |
| Estimated blood loss, mL | 118 ± 42 | 115 ± 39 | 0.564 |
| Urine output, mL | 405 ± 118 | 412 ± 121 | 0.649 |
| Vasopressor use, n (%) | 30 (23.44%) | 21 (18.26%) | 0.322 |
| Regional anesthesia/nerve block, n (%) | 86 (67.19%) | 81 (70.43%) | 0.586 |
| Intraoperative remifentanil dose, mg | 2.32 ± 0.23 | 2.29 ± 0.21 | 0.267 |
| Postoperative opioid consumption, morphine equivalents, mg | 34.8 ± 8.9 | 33.6 ± 8.3 | 0.278 |
| PONV prophylaxis, n (%) | 103 (80.47%) | 95 (82.61%) | 0.668 |
| Rescue antiemetic use, n (%) | 15 (11.72%) | 5 (4.35%) | 0.037 |
| Time to first mobilization, h | 19.3 ± 4.5 | 18.9 ± 4.3 | 0.479 |
| Overall ERAS adherence, % | 88.6 ± 5.9 | 89.1 ± 5.6 | 0.499 |

**Table footnote:**
ERAS: enhanced recovery after surgery; PONV: postoperative nausea and vomiting.
