# Supplementary material for: Application of remimazolam anesthesia in elderly patients undergoing radical resection for colorectal cancer: a cohort study on gastrointestinal recovery and complication rates
Source: Front Oncol. 2026 Jul 8;16:1867238. doi: 10.3389/fonc.2026.1867238 (PMC13388177; doi:10.3389/fonc.2026.1867238)
Supplement: Supplementary Table 2 — Association of Remimazolam with PONV and Postoperative Delirium After Propensity Score Matching. [file Table2.docx]

**Supplementary Table S2. Association of Remimazolam with PONV and Postoperative Delirium After Propensity Score Matching**

| **Outcome** | **Model** | **Covariates** | **OR for remimazolam vs propofol** | **95% CI** | **P value** |
| --- | --- | --- | --- | --- | --- |
| PONV | Crude logistic model based on matched cohort | None | 0.36 | 0.14–0.95 | 0.038 |
| Postoperative delirium | Crude logistic model based on matched cohort | None | 0.42 | 0.20–0.86 | 0.019 |
| PONV | Adjusted logistic regression | Age, sex, ASA classification, type of resection, intraoperative remifentanil dose, postoperative opioid consumption, antiemetic prophylaxis, rescue antiemetic use, ERAS adherence | To be calculated from raw data | To be calculated from raw data | To be calculated from raw data |
| Postoperative delirium | Adjusted logistic regression | Age, sex, ASA classification, documented baseline cognitive impairment, frailty-related information, postoperative opioid consumption, anticholinergic exposure, ICU admission, postoperative infection, hypoxemia, postoperative complications | To be calculated from raw data | To be calculated from raw data | To be calculated from raw data |
